# Income, health, and racial gaps between 340B hospitals, child sites, and nearby neighborhoods

**DOI:** 10.1093/haschl/qxaf121

**Published:** 2025-06-14

**Authors:** Neal Masia, Darren Filson, Silas Martin, Ulrich Neumann

**Affiliations:** Health Capital Group, LLC, Princeton, NJ 08540, United States; Department of Economics, Columbia University, New York, NY 10027, United States; Robert Day School of Economics and Finance, Claremont McKenna College, Claremont, CA 91711, United States; Scientific Affairs, Johnson & Johnson, Titusville, NJ 08560, United States; Scientific Affairs, Johnson & Johnson, Titusville, NJ 08560, United States

**Keywords:** 340B Drug Purchasing Program, health policy, hospital economics, health disparities

## Abstract

**Objectives:**

To estimate neighborhood differences between 340B child sites, parent hospital covered entities (CEs), and other neighborhoods near CEs.

**Methods:**

We created a unique dataset that contains CE and child site characteristics, and Zip Code Tabulation Area (ZCTA) socioeconomic and health data in 2022 for over 12 000 out-of-ZCTA code 340B hospital child sites. We computed differences across key measures, including median income, uninsured and unemployment rates, age, and health metrics between each pair and between the child site's ZCTA and all other ZCTAs within a 10-mile radius of the CE.

**Results:**

The median child-site ZCTA income is 28% higher than CE ZCTA income and approximately 11% higher than CE neighborhood ZCTA income. Uninsured rates (11% lower than CE ZCTA and 10% lower than CE neighborhood ZCTA) and unemployment rates (17% and 15% for CE ZCTA and CE neighborhood ZCTA, respectively) are lower in child-site areas and where the share of White residents is higher (11% and 9%, respectively). Average health status is better in child-site ZCTAs despite a higher median age.

**Conclusion:**

Our analysis suggests that 340B entities place child sites in neighborhoods that are wealthier, healthier, better insured, and less diverse than the neighborhoods of both the CE and other neighborhoods within a 10-mile radius of the CE.

## Introduction

The 340B Drug Pricing Program is a safety-net program that requires manufacturers to offer steep discounts on outpatient drugs to certain providers known as covered entities (CEs).^1^ Established in 1992, the program's limited objective was to restore outpatient drug discounts that manufacturers had given before enactment of the Medicaid rebate program to certain safety-net providers that directly purchased drugs and provided clinical care to a large number of uninsured patients.^2^ By statute, the minimum 340B discount equals the Medicaid discount, which incorporates a minimum 23.1% rebate, an inflation rebate, and the best commercial price offered by the manufacturer.

Covered entities earn significant profits by charging patients, their insurers, or Medicare standard rates for drugs acquired at deeply discounted 340B acquisition costs. The CEs distribute purchased medicines directly or through contract pharmacies (CPs). The CEs may pass 340B discounts to underserved patients, but they are not required to do so. Alternatively, CEs may use 340B profits to support patient care indirectly—for example, by investing in community health initiatives. There are no binding requirements on how (or whether) CEs must deploy 340B funds.

Over 2500 nonprofit hospitals are now CEs, reflecting 340B's exponential growth.^3,4^ Prior research has estimated that 340B profits exceeded $40 billion in 2019,^5^ and recent estimates suggest that the aggregate amount of 340B discounts was $58 billion in 2023,^6^ with over $66 billion of inventory acquired at the 340B price.^7^ Recent research has found that at least 75% of 340B income flows to CPs or is kept by CEs as retained earnings, and that 340B hospitals have lower levels of charity spending.^8^

The 340B hospital CEs may register “child sites,” including clinics, infusion centers, and various other outpatient treatment centers, that may be located on the hospital's main campus or remotely. Child sites must be a reimbursable clinic on the CE's Medicare cost report but otherwise face few limitations.^9^ There were over 34 000 340B hospital child sites in 2023, up from just over 7000 in 2013.^10^ The proliferation of hospital-owned, 340B-affiliated clinics could have the potential to increase access for lower-income patients, although factors unrelated to the program may impact the optimal location for any health care clinic.

How do 340B hospitals choose where to put child sites? If child sites are in lower-income areas relative to the CE, they may directly fulfill the 340B's mission of increasing access to high-quality care for low-income patients. If child sites are instead placed in higher-income areas, they might focus on serving better-insured patients while generating additional income for the CE. Since these profits can be used in various ways, fulfilling the 340B mission hinges on accountability to ensure they are indirectly used to enhance services for vulnerable patients. Understanding the locations of child sites and how profits from child sites are used can offer policymakers valuable insights when considering potential adjustment to the 340B program to ensure that activities and incentives align with the mission of assisting patients in need.

Anecdotal reports have identified instances where hospitals serving low-income patients have opened clinics in higher-income areas, sometimes within the walls of hospitals ineligible for 340B that serve mainly higher-income patients.^11^ Recent reviews of peer-reviewed literature highlight differences in strategic behavior among various types of CEs.^12,13^ The evidence indicates that nonprofit, disproportionate share hospitals (DSH) appear to leverage the program in “margin-motivated” ways.^12^ In contrast, similarly rigorous studies suggest some limited evidence of “mission-driven” behavior.^12^

Systematic research has examined differences in Zip Code Tabulation Area (ZCTA) income levels between CEs and CPs^14^ and income characteristics of CP locations generally.^15^ In 2014, Conti and Bach^16^ examined differences in income and insurance coverage as of 2012 between different types of hospitals and child sites, finding significant differences between hospital and child-site locations and showing that child sites are located in higher-income areas. In 2012, however, there were fewer than 1000 hospitals in the program, and just a few thousand child sites. Another study examining child sites and contract pharmacies in 2021 also found differences in income and racial composition between parent entity and child-site locations.^17^

## Data and methods

We extracted data on all active 340B CEs and child sites as of 2022 from the Health Resources and Services Administration (HRSA) Office of Pharmacy Affairs (OPAIS) database.^18^ The data include the type of CE (e.g., DSH, critical access hospital, etc.) and ZIP code of the hospital and the child site. We converted ZIP codes (with a minimum population of 1000) to ZCTAs using the Centers for Medicare and Medicaid Services (CMS) Uniform Data System (UDS) data mapper.^19^ Of the total 22,069 child sites in the dataset, approximately 43% were identified as being either on the premises of their parent CE or located within the same ZCTA. We analyzed 12 632 CE–child-site pairs where the child site was in a different ZCTA than the parent CE (57% of the 22 069 total child sites in our data). We calculated the distance between CE and child sites using the ZCTAs’ corresponding latitude and longitude coordinates.^20^

For each child-site–CE pair, we tabulated variables in 3 places: the child-site ZCTA, the CE ZCTA, and the average across ZCTAs within 10 miles of the CE. Variables included median household income, unemployment rate, uninsured rate, share of ZCTA population identifying as White, and the adult diabetes, stroke, smoking, and obesity rates. Income, insurance status, and uninsured rates were collected from the 2022 American Community Survey,^21^ and health data came from the Centers for Disease Control and Prevention PLACES database.^22^ We selected metrics that were broadly available and are known areas of potential health disparities across socioeconomic groups. We examined the difference between child-site and CE ZCTA levels across our list of variables and separately examined the difference between child-site and 10-mile-radius ZCTAs.

## Results

**Figure 1.**
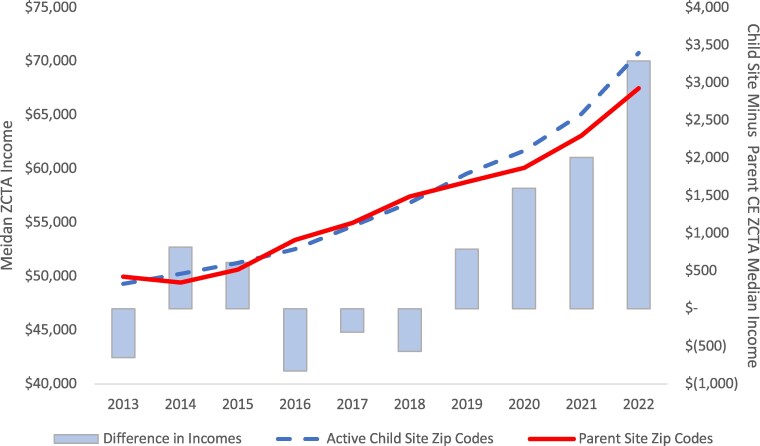
Child site and parent hospital CE ZCTA income levels, growth and differences, 2013–2022. Dashed line indicates child site ZCTA median household income (in US dollars). Solid line indicates CE ZCTA median income. Bars depict differential between child-site and CE ZCTA income levels. Abbreviations: CE, covered-entity; ZCTA, Zip Code Tabulation Area.

Figure 1 shows the growth in median household income across active child-site and parent CE ZCTAs from 2013 through 2022. While average parent CE ZCTA income grew by 3.4% per year over that period, child-site entity ZCTA grew at a faster 4.1% annual rate. This dynamic resulted in a growing gap between child-site and CE income levels overall but does not separate child sites that are co-located with the CE from other child sites located in other ZCTAs.

Table 1 shows the difference between child site and CE ZCTAs as well as nearby neigborhood ZTCAs across various socioeconomic and health metrics as of 2022. The median household income was over 28% higher in child-site ZCTAs than in parent CE ZCTAs. Unemployment and uninsured rates were 17% and 11% lower in the child-site areas, respectively. Despite a higher median resident age (+3.1 years), child-site areas had lower rates of adult diabetes (−1.2%), stroke (−3.2%), obesity (−3.2%) and smoking (−7.1%).

**Table 1.** Differences between covered-entity, child-site, and nearby ZCTAs, 2022.

	Child-site ZCTA compared with CE ZCTA				Child-site ZCTA compared with ZCTAs within 10 miles of CE			
	CE ZCTA average	Average child-site difference	Average % difference	Share with same direction difference	Other ZCTAs (near CE) average	Average child-site difference	Average % difference	Share with same direction difference
Household income	$60 743	+$17 008	+28%	70%	$71 714	+$8032	+11%	60%
Unemployment rate	6.4%	−1.1%	−17%	64%	6.1%	−0.9%	−15%	66%
Uninsured rate	11.4%	−1.2%	−11%	63%	11.3%	−1.1%	−10%	66%
Share of White residents	63%	+7%	+11%	62%	64%	+6%	+9%	61%
Adult diabetes rate	12.0%	−0.1%	−1.2%	50%	12.3%	−0.4%	−3.2%	57%
Adult stroke rate	3.68%	−0.1%	−3.2%	49%	3.61%	−0.2%	−4.4%	58%
Adult smoking rate	15.4%	−1.1%	−7.1%	57%	15.7%	−1.1%	−7.0%	62%
Adult obesity rate	35.2%	−1.1%	−3.2%	55%	35.2%	−1.1%	−3.2%	61%
Median resident age (years)	36.2	+3.1	+8.6%	65%	39.6	+1.4	+3.5%	60%

Abbreviations: CE, covered-entity; ZCTA, Zip Code Tabulation Area.

We also examined whether these differences would occur if 340B hospitals are located in particularly poor areas by happenstance. Over 50% of child sites situated in a different ZCTA are located within a 10-mile radius of the parent CE. The mean hospital CE had 20.1 ZCTAs within a 10-mile radius, which indicates that CEs had considerable alternative options for child site placement. The average CE could choose to locate the average child site well within a 10-mile radius, so the choice of child-site location likely reflects a meaningful decision by the CE. We calculated the “nearby neighborhood” average across all ZCTAs within 10 miles of the parent entity to compare the location of the chosen child site with potential alternative ZCTAs. Results were similar with smaller and larger radius choices. Compared with alternative site choices, child sites were located in areas with significantly higher income (+11%), lower unemployment (-15%), fewer uninsured people (-10%), and a higher proportion of White residents (+9%). Despite a higher median resident age (+1/4 years), child-site areas had lower rates of adult diabetes (−3.2%), stroke (−4.4%), obesity (−3.2%), and smoking (−7.0%).

## Discussion

This analysis suggests that it is much more likely for 340B hospital CEs to place child sites in locations with relatively more affluent patients with lower uninsured and unemployment rates than both the CE's ZCTA and the CE neighborhood's ZCTA. Differences in average health measures were not as large but followed the same direction. For every 10 000 people in a ZCTA, the health measures suggest that there were hundreds fewer residents in worse health compared to those areas into which hospitals expanded.

In interpreting these results, we acknowledge that there are multiple potential motivations for CEs to target affluent, well-insured neighborhoods for child-site expansion. In view of the literature on 340B program incentives, maximizing 340B profits is one likely explanation—but our analysis cannot separate it from others, such as an inherent interest in placing clinics in well-insured areas to optimize access to well-insured patients, location, competition, etc. The opportunity to earn higher 340B profits could simply be incidental to the broader strategy of hospital revenue and business optimization. One challenge for our study is the lack of a comprehensive data source on non-340B child sites, which prevents a comparison of their locations to those of 340B-affiliated child sites.

Regardless of the reason, child sites placed in neighborhoods already well-served by the health system might exacerbate health disparities between communities and work against the 340B goal of expanding access in areas with greater need. This does not preclude potential indirect benefits, as hospital activities generating profits from these areas could advance mission-driven goals by supporting hospital-wide improvements or by facilitating the indirect use of 340B gains for community health initiatives. But without transparent and systematic tracking of such activities, no analysis can address whether hospitals reinvest 340B profits. This lack of accountability creates uncertainty regarding the impact of child-site placement, as it remains unclear whether program expansion effectively advances the program's goals to serve vulnerable patients. The absence of standardized mandatory or systematic voluntary reporting has historically limited researchers in empirically confirming the program's benefits to patients on a larger scale. Policymakers have thus debated whether to introduce transparency requirements or restrictions on how 340B profits are used to ensure that the program is resulting in measurable improvements for patients.

## Conclusion

Even at discounted prices, the 340B program is now larger than all federal drug programs except for Medicare Part D. Recent academic studies and government analyses document that 340B profits are directly and indirectly paid by manufacturers, employers, and taxpayers.^23-26^ The size, scope, and ongoing implementation questions inherent to 340B^27^ suggest that policymakers have a keen interest in understanding where 340B clinics are located given the implications for employers and taxpayers.

Our findings suggest that 340B child-site expansion creates profits for hospitals by targeting wealthier neighborhoods with better reimbursement prospects. Covered entities do not appear to increase care directly by placing sites closer to vulnerable patients in lower-income, higher-need neighborhoods. 340B profits may be used to achieve those goals indirectly, but better data are needed to guide policymakers on whether this is the case. Increased transparency could help in determining whether the benefits of 340B are ultimately flowing to patients who struggle to access the health system.

## Supplementary Material

qxaf121_Supplementary_Data

## References

[1] Health Resources and Services Administration . 340B Drug Pricing Program. Published 2024. Accessed December 15, 2024. https://www.hrsa.gov/opa

[2] Health Resources and Services Administration . 340B Drug Pricing Program. Published April 21, 2017. Accessed May 1, 2025. https://www.hrsa.gov/opa

[3] Avalere . 340B purchase data highlights continued program growth. October 2024. Accessed May 1 2025. https://advisory.avalerehealth.com/insights/340b-purchase-data-highlightscontinued-program-growth

[4] Health Resources and Services Administration . 340B Drug Pricing Program. Accessed December 15, 2024. https://www.hrsa.gov/opa

[5] Fein AJ . The 340B Program reached $66 billion in 2023—up 23% vs. 2022: analyzing the numbers and HRSA’s curious actions. Drug Channels. Accessed May 1, 2025. https://www.drugchannels.net/2024/10/the-340b-program-reached-66-billion-in.html

[6] Martin R, Karne H. The 340B Drug Discount Program grew to $124 billion in 2023. IQVIA Institute White Paper. 2024. Accessed May 1, 2025. https://www.iqvia.com/-/media/iqvia/pdfs/us/white-paper/2024/iqvia-update-on-size-of-340b-program-report-2024.pdf

[7] Health Resources and Services Administration . 2023 340B Covered Entity purchases. Accessed December 20, 2024. https://www.hrsa.gov/opa/updates/2023-340b-covered-entity-purchases

[8] Masia N. Measuring the 340B Drug Purchasing Program's impact on charitable care and operating profits for covered entities. Health Capital Group White Paper. 2022. Accessed May 1, 2025. https://www.healthcapitalgroup.com/340b-profits-and-charity-care

[9] Health Resources and Services Administration . 340B Program FAQs. Accessed December 20, 2024. https://www.hrsa.gov/opa/faqs

[10] Office of Pharmacy Affairs . 340B OPA Information System. Health Resources and Services Administration. Accessed December 15, 2024. https://340bopais.hrsa.gov/CoveredEntitySearch/000125701

[11] Thomas K, Silver-Greenberg J. Profits over patients: how a hospital chain used a poor neighborhood to turn huge profits. The New York Times. Published September 24, 2022. Updated September 27, 2022. Accessed December 15, 2024. https://www.nytimes.com/2022/09/24/health/bon-secours-mercy-health-profit-poor-neighborhood.html

[12] Levengood TW, Conti RM, Cahill S, Cole MB. Assessing the impact of the 340B drug pricing program: a scoping review of the empirical, peer-reviewed literature. Milbank Q. 2024;102(2):429–462. 10.1111/1468-0009.12691 38282421 PMC11176403

[13] Knox RP, Wang J, Feldman WB, Kesselheim AS, Sarpatwari A. Outcomes of the 340B drug pricing program: a scoping review. JAMA Health Forum. 2023;4(11):e233716. 10.1001/jamahealthforum.2023.3716 37991784 PMC10665972

[14] Masia N, Kuwonza F. Income differences between locations of 340B entities and contract pharmacies. Am J Manag Care. 2023;29(6):e184–e188. 10.37765/ajmc.2023.89377 37341983

[15] Nikpay S, Gracia G, Geressu H, Conti R. Association of 340B contract pharmacy growth with county-level characteristics. Am J Manag Care. 2022;28(3):133–136. 10.37765/ajmc.2022.88840 35404549

[16] Conti RM, Bach PB. The 340B Drug Discount Program: hospitals generate profits by expanding to reach more affluent communities. Health Aff (Millwood). 2014;33(10):1786–1792. 10.1377/hlthaff.2014.0540 25288423 PMC4591849

[17] Avalere Health . Accessed May 1, 2025. https://avalere.com/insights/340b-hospital-child-sites-and-contract-pharmacy-demographics

[18] US Department of Health and Human Services . Health Resources and Services Administration Office of Pharmacy Affairs 340B OPAIS. Accessed September 22, 2021. https://340bopais.hrsa.gov/CoveredEntitySearch/000125701

[19] HUDUser . Accessed December 15, 2024. https://www.huduser.gov/portal/datasets/usps_crosswalk.html

[20] United States Census Bureau. All US ZCTAs with their corresponding latitude and longitude coordinates. http://www.census.gov/geo/maps-data/data/gazetteer.html. Gist. Accessed October 8, 2021. https://gist.github.com/erichurst/7882666

[21] United States Census Bureau . 2022 American Community Survey 1-Year Estimates. Accessed December 15, 2024. https://www.census.gov/programs-surveys/acs.html

[22] Centers for Disease Control and Prevention . PLACES: Local Data for Better Health. Published 2024. Accessed December 15, 2024. https://www.cdc.gov/places/

[23] Masia N, Motyka J, Westrich K, Campbell J. The 340B Program and commercial insurance premiums. Health Capital Group White Paper, 2025, and Masia, N. 340B and Overall Medicaid Spending. Health Capital Group White Paper. 2024. Accessed December 24, 2024. https://www.healthcapitalgroup.com/340b-and-total-medicaid

[24] State of North Carolina Treasurer. State Treasurer Folwell Finds North Carolina 340B Hospitals Overcharged State Employees for Cancer Drugs, Reaped Thousands of Dollars in Profits Per Claim. 2024. Accessed December 15, 2024. https://www.nctreasurer.com/news/press-releases/2024/05/08/state-treasurer-folwell-releases-report-finding-north-carolina-340b-hospitals-overcharged-state

[25] Martin R, Illich K. Are discounts in the 340B Drug Discount Program being shared with patients at contract pharmacies? IQVIA. Accessed May 1, 2025. https://www.iqvia.com/-/media/iqvia/pdfs/us/white-paper/are-discounts-in-the-340b-drug-discount-program-being-sharedwith-patients-at-contract-pharmacies.pdf

[26] Masia N, Motyka J, Westrich K, Campbell J. The 340B program and commercial insurance premiums. Health Capital Group White Paper. 2025. Accessed June 5, 2025. www.healthcapitalgroup.com

[27] Mulligan K . The 340B Drug Pricing Program: background, ongoing challenges and recent developments. USC—Schaeffer Center White Paper, October 14, 2021. Accessed September 15, 2022. https://healthpolicy.usc.edu/research/the-340b-drug-pricing-program-background-ongoing-challenges-and-recent-developments/

